# Supplementary material for: Removal of Toxic and Essential Nutrient Elements from Commercial Rice Brands Using Different Washing and Cooking Practices: Human Health Risk Assessment
Source: Int J Environ Res Public Health. 2022 Feb 23;19(5):2582. doi: 10.3390/ijerph19052582 (PMC8909527; doi:10.3390/ijerph19052582)
Supplement: Supplementary file 1 [file ijerph-19-02582-s001.zip › ijerph-1538948-supplementary.pdf]

Supplementary Information

Removal of toxic and essential nutrient elements from commercial rice brands using different washing and cooking practices: Human health risk assessment

Syfullah Shahriar[1,2*] • Alok Kumar Paul[2] • Mohammad Mahmudur Rahman[1,*]

[1]Global Centre for Environmental Remediation (GCER), College of Engineering, Science and Environment, The University of Newcastle, Callaghan, NSW 2308, Australia

[2]Department of Soil Science, Sher-e-Bangla Agricultural University, Dhaka 1207, Bangladesh

*Corresponding author

E-mail address: mahmud.rahman@newcastle.edu.au (M.M. Rahman); Syfullah.shahriar@uon.edu.au (S. Shahriar)

Digestion and analysis of food samples

Briefly, powdered rice samples (0.5 g) were weighed and placed into prewashed 100 ml glass digestion tubes and 5 ml concentrated nitric acid (trace analytical grade, 70%, Fisher Chemical) was added into each tube and the solution mixtures were placed inside a fume hood overnight. The tubes were heated for 5 to 6 hours at 140 ºC temperature in a block Digester (Seal BD 50) until only a few ml of residual solutions were left in the tubes. Following digestion, tubes were removed and allowed to cool at room temperature. Once cool, samples were diluting to 10 ml with 0.1% nitric acid solution. The diluted samples were mixed thoroughly with a vortex mixer and then filtered using a 0.45µm syringe filter (cellulose acetate, Minisart) into 10 ml plastic tubes. The diluted samples were stored in a refrigerator at 4ºC until analysed by the inductively coupled plasma mass spectrometry (ICP-MS, 7900, Agilent Technologies, Japan) for TEs and nutrient elements.

Health risk assessment

To assess the non-carcinogenic human health risks from the exposure of TEs, we used US EPA hazard quotient (HQ) [49]. The HQ value is the ratio of estimated daily intake of a TE to its oral reference dose (Rfd).

$$\mathrm{HQ} = \frac{\mathrm{EDI}}{\mathrm{Rfd}}$$

The RfDs of As, Cd and Pb were 0.3, 1 and 12.5 (µg/kg/day), respectively [50,51]. HQ values ≤ 1 indicates no obvious risk from the TEs to the exposed population over a lifetime exposure, while HQ values > 1 depicts the chance of occurrence of non-carcinogenic risk.

The incremental lifetime cancer risk (ILCR) from ingestion of TEs was calculated using the following equation:

$$\mathrm{ILCR} = \mathrm{EDI} \times \mathrm{SF}.$$

Where SF is the oral slope factor of the TEs. The slope factor for As, Cd and Pb are 1.5, 0.38 and 8.5×10^{-3} mg/kg/day [52-54].

Table S1. Grain characteristics of selected brands of rice.

Rice Variety	Grain characters
BR 22	Grain: short, bold, translucent
Banshful	Aromatic (grain: coarse, short)
Hybrid Heera 1	Salt tolerant (grain: medium coarse, short)
BRRIdhan 50	Aromatic, protein content 8.2% (grain: long slender, white)
BRRIdhan 28	High yielding (grain: medium slender, white)
Paijam	Aromatic (grain: fine)
Jirashail	Grain: fine, slender, white
BRRIdhan 34	Aromatic (grain: white, short, bold)
Binni rice	Aromatic (grain: opaque, sticky)
Katarivogh	Aromatic (grain: fine)

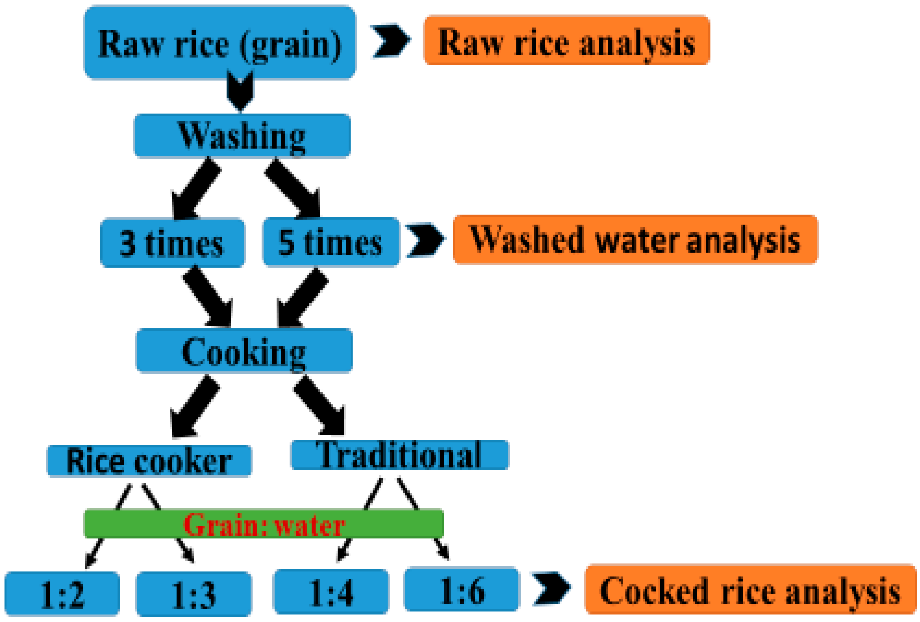

Figure S1. Flow chart of washing and cooking procedures

Table S2. Concentrations (μg/kg) of TEs and nutrient elements in SRMs.

	SRM1568b (Rice flour)			SRM1573a (Trace elements in tomato leaves)			SRM1570a (Trace elements in spinach leaves)		
Elements	Certified value	Observed value	% Recovery	Certified value	Observed value	% Recovery	Certified value	Observed value	% Recovery
As	285 ± 14	311 ± 7	109	112.6 ± 2.4	108.4 ± 5.2	96.2	68 ± 12	63 ± 9	92.6
Cd	22.4 ± 1.3	21.2 ± 1.1	94	1517 ± 27	1430 ± 68	94.3	2876 ± 58	2692 ± 88	93.6
Pb	-	27 ± 8	-	-	1390 ± 60	-	200*	230 ± 18	115
Zn	19420 ± 260	17113 ± 117	88	30940 ± 550	28330 ± 840	91.5	82300 ± 3900	77600 ± 5400	94.3
Cu	2350 ± 160	2197 ± 11	93	4700 ± 140	5100 ± 210	108.5	12220 ± 860	13130 ± 1080	107.4
Mn	19200 ± 1800	18210 ± 207	94	246300 ± 7100	248860 ± 9200	101.0	76000 ± 1200	68900 ± 1560	90.6
Se	365 ± 29	398 ± 24	109	54.3 ± 2.0	49.7 ± 6.3	91.5	115.2 ± 4.3	109.6 ± 5.4	95.1
Mo	1451 ± 48	1220 ± 31	84	460*	390 ± 20	84.8	-	408.5 ± 10.2	-

*information only
